# Pharmacological Inhibition of Cav2.2 Channels by Recombinant Phα1β Toxin (CTK01512‐2)

**DOI:** 10.1111/bcpt.70268

**Published:** 2026-07-01

**Authors:** Natália Virtude Carobin, Thamyris Reis Moraes, Luana Assis Ferreira, Christopher Kushmerick, Marcus Vinícius Gomez, Célio José Castro Junior

**Affiliations:** ^1^ Faculdade de Saúde Santa Casa BH Belo Horizonte Minas Gerais Brazil; ^2^ Psychological & Brain Science Indiana University Bloomington Indiana USA; ^3^ Departamento de Fisiologia Universidade Federal de Sergipe São Cristóvão Sergipe Brazil

**Keywords:** Cav2.2 channels, CTK01512‐2, neuropathic pain, *Phoneutria nigriventer*, Phα1β

## Abstract

Phα1β is a peptide toxin originally isolated from the venom of the Brazilian spider *Phoneutria nigriventer* , known for its potent analgesic effects in preclinical models of acute and chronic pain. However, clinical translation has been limited by production constraints. A recombinant analogue, CTK01512‐2, was developed and has demonstrated antinociceptive efficacy comparable to the native toxin in diverse experimental models. In this study, we examined the effects of CTK01512‐2 on N‐type voltage‐gated calcium channels (Cav2.2) expressed in transiently transfected HEK‐293 cells and confirmed its antinociceptive action in a mouse model of neuropathic pain. CTK01512‐2 induced a partial (~50%) and reversible inhibition of Cav2.2‐mediated calcium currents, with a calculated half‐maximal inhibitory concentration (IC_50_) of 3.94 nM. In contrast to ω‐conotoxin MVIIA, a clinically approved irreversible Cav2.2 blocker, CTK01512‐2 allowed current recovery after washout, indicating reversible binding. Notably, the toxin did not significantly alter the voltage dependence or kinetics of channel activation. These findings identify CTK01512‐2 as a selective and reversible Cav2.2 channel modulator, underscoring its therapeutic potential as a novel analgesic agent for chronic pain management.

## Plain Language Summary

Phα1β is a peptide from the venom of the Brazilian spider *Phoneutria nigriventer* that has shown strong pain‐relieving effects in experimental studies. Because producing the native toxin is challenging, a recombinant version (CTK01512‐2) was developed. In this study, we investigated how CTK01512‐2 acts on N‐type calcium channels (Cav2.2), which play a key role in transmitting pain signals. The compound partially and reversibly blocked these channels in human cells grown in the laboratory and reduced pain behaviour in mice with neuropathic pain. Unlike some existing channel blockers, its effects were reversible. These findings support CTK01512‐2 as a promising candidate for chronic pain treatment.

## Graphical abstract

The recombinant Phα1β toxin (CTK01512‐2) partially and reversibly inhibits Cav2.2 calcium channels in HEK‐293 cells. CTK01512‐2 displays dose‐dependent inhibition of N‐type calcium currents, with an IC_50_ of 3.94 nM. In vivo, CTK01512‐2 effectively reduces mechanical allodynia in a neuropathic pain model, confirming its antinociceptive activity.

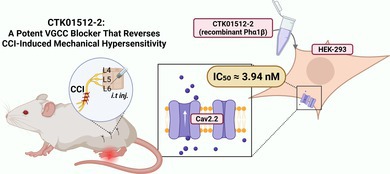

## 1 Introduction

Phα1β neurotoxin is a 55‐amino acid peptide containing six disulfide bridges, previously known as Tx3–6 [1]. It was originally purified from the venom of the Brazilian spider *Phoneutria nigriventer* and has demonstrated potent analgesic efficacy in rodent models of both acutely and chronically induced pain [2, 3, 4]. However, the clinical translation of this promising molecule has been hindered by scalability issues, primarily due to the difficulty of isolating sufficient quantities directly from the venom. Its structural complexity poses a major challenge for chemical synthesis, and efforts to date have failed to produce a biologically active analogue with efficacy comparable to the native toxin. Our group recently developed a recombinant analogue of Phα1β, designated CTK01512‐2, produced via heterologous expression in *Escherichia coli* . This recombinant peptide has demonstrated robust analgesic‐like effects across a range of experimental pain models, including acute and spontaneous pain, inflammatory pain, neuropathic pain, visceral pain and cancer‐related pain [3, 5]. In these preclinical studies, CTK01512‐2 showed comparable efficacy and potency to the native Phα1β toxin. Given its enhanced availability, CTK01512‐2 has also been tested and demonstrated therapeutic effectiveness in preclinical models not yet tested with the native toxin, such as pancreatitis‐induced pain [6], orofacial pain [7] and diabetic neuropathic pain [8]. Moreover, CTK01512‐2 has exhibited antinociceptive activity following intravenous administration [9, 10], a finding highly relevant for its potential clinical development.

Despite its established analgesic properties, the precise molecular mechanism by which CTK01512‐2 reduces nociception is still under investigation. Previous studies have shown that native Phα1β inhibits high‐voltage‐activated (HVA) calcium channels, with marked selectivity for Cav2.2 N‐type calcium channels [11]. These channels play a critical role in the transmission of nociceptive signals by regulating glutamate release from primary sensory neurons at synapses in the dorsal horn of the spinal cord. Inhibition of Cav2.2 channels can suppress nociceptive input to the central nervous system, ultimately resulting in analgesia [12, 13]. Modulation of these channels is of therapeutic interest in the context of chronic pain [14]. Conversely, Ziconotide is a clinically approved analgesic which derives from ω‐conotoxin MVIIA, a peptide that selectively blocks Cav2.2 channels [15, 16]. To date, it has not been determined whether CTK01512‐2 engages the same molecular targets as native Phα1β. Hence, the objective of the present study is to investigate the effects of CTK01512‐2 on voltage‐gated calcium currents mediated by Cav2.2 (N‐type) channels in HEK293 cells transiently transfected with these channels.

## 2 Material and Methods

### 2.1 Animals

The study was conducted in accordance with the Basic & Clinical Pharmacology & Toxicology policy for experimental and clinical studies [17]. For behavioural tests, we used male Swiss mice aged 8 weeks. Mice were kept in polypropylene cages (290 × 220 × 140 mm) housing up to six animals each, with water and food provided ad libitum, and maintained on a 12 h dark–light cycle at 22°C–24°C with a relative humidity of 50% ± 5%. The study was previously approved by the Santa Casa Ethics Committee for the Experimental Use of Animals (CEPEEA, protocol number 003/2018) and was conducted in accordance with the IASP Guidelines for the Use of Animals in Research. Thirty‐six animals were used in this study and were randomly allocated into six groups as described in the results section.

### 2.2 Mechanical Nociceptive Threshold Measure

To assess the nociceptive threshold, the von Frey filament test was used. For this test, animals were acclimatized for at least 60 min in boxes placed over a metal platform. The floor of the boxes consisted of a small metal grid that allowed access to the plantar surface of each animal's paws. von Frey filaments of increasing thicknesses, corresponding to 0.07, 0.16, 0.4, 0.6, 1.0, 1.4 and 2.0 g‐force, were applied to the plantar surface of the right paw (ipsilateral) until a withdrawal reflex was evoked. The test was performed three times, and the mean response was recorded as the nociceptive threshold [18].

### 2.3 Chronic Constriction Injury (CCI)

Neuropathic pain was induced using the CCI model of the sciatic nerve, as previously described [19]. Mice were deeply anaesthetised with isoflurane (2%, delivered by inhalation), and the common sciatic nerve was exposed at mid‐thigh level through blunt dissection. Three loose ligatures of 4–0 silk thread were placed around the nerve, proximal to its trifurcation, with approximately 1 mm spacing between them. Care was taken to avoid completely occluding the nerve or disrupting blood flow. Sham‐operated animals underwent the same surgical procedure, except that the sciatic nerve was exposed but not ligated. Under our experimental conditions, all of the CCI animals showed a drop of mechanical threshold to below 0.4 gf after CCI surgery, and there was no exclusion of animals for the analysis.

### 2.4 Intrathecal Injection

Both CTK01512‐2 (200 pmol/site) and MVIIA were injected intrathecally. For this, the animals were anaesthetised with isoflurane (2%) and positioned in prone position. Each drug was injected into the L5–L6 intervertebral segments, in a volume of 5 μL using a 10‐μL syringe (Model 701N, Hamilton, USA). The experimenter was blinded to the drug injected to the animals as well as to the mechanical nociceptive threshold measurements by means of previous codification of the drug‐flasks and animals.

### 2.5 Cell Culture

Human embryonic kidney cells (HEK‐293) were cultured in 100 × 20 mm culture dishes with standard DMEM (Dulbecco's modified Eagle medium) supplemented with 10% foetal bovine serum (FBS), 50 U/mL penicillin and 50 μg/mL streptomycin. The cells were maintained in a humidified atmosphere of 95% O_2_ and 5% CO_2_ at 37°C. Cells were passaged every 3 days.

### 2.6 Cell Transfection

HEK cells were grown to approximately 80% confluence, enzymatically dissociated with 0.25% trypsin, plated in 6‐well plates (10 cm^2^ surface area), and allowed to recover for 48 h. Cells were transiently transfected using Lipofectamine, following the manufacturer's instructions, in Opti‐MEM medium with cDNA plasmids encoding the N‐type calcium channel (Cav2.2), including the α1b (Addgene: 26569), β3 (Addgene: 26574) and α2δ (Addgene: 26575) subunits together with eGFP (Clontech. CA, USA). To achieve optimal transfection efficiency while minimizing cytotoxicity, 1 μg of cDNA was used with 2.5 μL of Lipofectamine. Cells were incubated at 37°C in a 95% O_2_ and 5% CO_2_ atmosphere for 12 to 18 h before electrophysiological recordings.

### 2.7 Toxins and Reagents

Giotto Biotech (https://www.giottobiotech.com) synthesized the recombinant version of Phα1β via *Escherichia coli* expression for evaluation. It was purified through a proprietary production process that combined ion exchange and size exclusion chromatography. The final yield of the process was 0.5 mg/mL, and the purity of the recombinant toxin is higher than 90%. The peptide molecular weight (*M*_w_) was 6.045 kDa. Both native and the recombinant Phα1β share the same 55 amino acid sequence:

ACIPRGEICTDDCECCGCDNQCYCPPGSSLGIFKCSCAHANKYFCNRKKEKCKKA

The amino acid sequence of the recombinant peptide is identical to that of native Phα1β, except for the addition of a methionine at the N‐terminal portion of the recombinant peptide. ω‐conotoxin MVIIA was obtained from Latoxan (Valence, France), and cadmium was provided by Sigma‐Aldrich (USA). Stock solutions of the toxin were prepared in PBS and stored in siliconized plastic tubes (Eppendorf). All solutions were stored at −20°C and diluted to the desired concentrations immediately before use.

### 2.8 Electrophysiological Recordings

Standard whole‐cell voltage‐clamp recordings in HEK293 cells were performed as described previously [20, 21]. Recordings were performed with an Axopatch 200B amplifier (Molecular Devices, USA) and digitized using an Axon Digidata 1200 series analogue‐to‐digital converter (Axon Instruments, USA), controlled by the pClamp version 8.2 (Axon Instruments, USA). The amplified currents were low‐pass filtered at 2 kHz and sampled at > 5 kHz. The external solution for all recordings contained (in mM) 1 CaCl_2_, 4 MgCl_2_, 10 HEPES and 135 TEA‐Cl; pH was adjusted to 7.2 with CsOH. Internal solution contained (in mM) 126 CsCl, 10 Cs‐EGTA, 1 Cs‐EDTA, 10 HEPES and 4 Mg‐ATP, pH 7.2 with CsOH. Pipettes with resistances ranging from 2.0 to 4.0 MΩ were fabricated from glass capillary tubes (Patch Glass, PG150T‐7.5, Warner Instruments) using a two‐stage vertical pipette puller (PP‐830, Narishige, Tokyo, Japan). Pipettes were coated with dental wax to within approximately 0.1 mm of the tip to reduce electrical capacitance. Series resistances (< 6 MΩ) were compensated 70%–80% with a 10‐μs lag time. We evoked calcium currents by voltage steps and leak‐subtracted currents online using a P/‐4 protocol. Cells were plated on 30 × 15 mm dishes, and single cells were selected based on eGFP fluorescence. Each cell was clamped with a microelectrode‐filled pipette containing internal solution. Upon obtaining a high‐resistance seal (> 1GΩ), leak currents < 20pA, and rupturing the membrane to achieve whole‐cell configuration, the cells were typically held at −80 mV. Cells not meeting these criteria were excluded from analysis.

For analysis of N‐type calcium channel antagonism, cells were perfused with Phα1β toxin at concentrations of 5, 25, 125 and 625 nM, diluted in external solution. All recordings were conducted at room temperature (22°C–25°C). To evoke voltage‐activated currents, cells were stimulated from a holding potential of −80 to 0 mV for 40 ms every 7 s under control conditions (without toxin) and after toxin perfusion as indicated. We applied toxin‐containing solutions with their flows controlled by computer‐driven solenoids (Warner instruments, VC‐66CS) coupled to a microperfusion system using fine glass pipettes placed in close proximity to the cell in order to reduce dead time to within 1 s.

### 2.9 Statistical Analysis

Data are presented as mean ± standard error of the mean (SEM). When appropriate, values were normalized to the percentage of the control group. Comparisons of inhibition percentages in current–voltage (*I*–*V*) relationship experiments were performed using a paired *t* test. Behavioural data were analysed using two‐way ANOVA followed by the appropriate post hoc tests. For comparisons between two experimental groups, Sidak's multiple comparisons test was applied. For analyses of compound efficacy across time points, Dunnett's post hoc test was used to compare each treatment group with the CCI‐vehicle–treated group. In order to verify the assumption of normality we performed the Shapiro–Wilk test on data sets. Groups with *p* > 0.05 were considered to follow a normal distribution and were analysed using parametric tests. All statistical analyses and graph generation were performed using GraphPad Prism, version 10 (GraphPad Software, La Jolla, CA, USA). For the dose response analysis, a Log(agonist) versus response equation was used (Y = Bottom + (Top − Bottom)/($1+10^{\left(\mathrm{Log}{\mathrm{EC}}_{50}-X\right)}$)) with bottom constraint at 0 and Top set as the maximum observed effect. We have assumed a standard slope (hillslope = 1). The activation state of N‐type Ca^2+^ channels were examined by fitting activation curves to the *I*–*V* relationships of each experimental group. A nonlinear regression model was used to estimate the reversal potential (*E*_rev_), based on points near the intersection with the *X*‐axis. Conductance values were calculated from *E*_rev_ and normalized to the maximum conductance (*G*/*G*_max_). The resulting activation curves were fitted with a sigmoidal function to determine the half‐maximal activation potential (*V*_h_) and the slope factor (*k*), which describes the steepness of the activation curve.

## 3 Results

### 3.1 Effects of Phα1β Toxin and MVIIA on Mechanical Allodynia in the CCI Model

**FIGURE 1:**
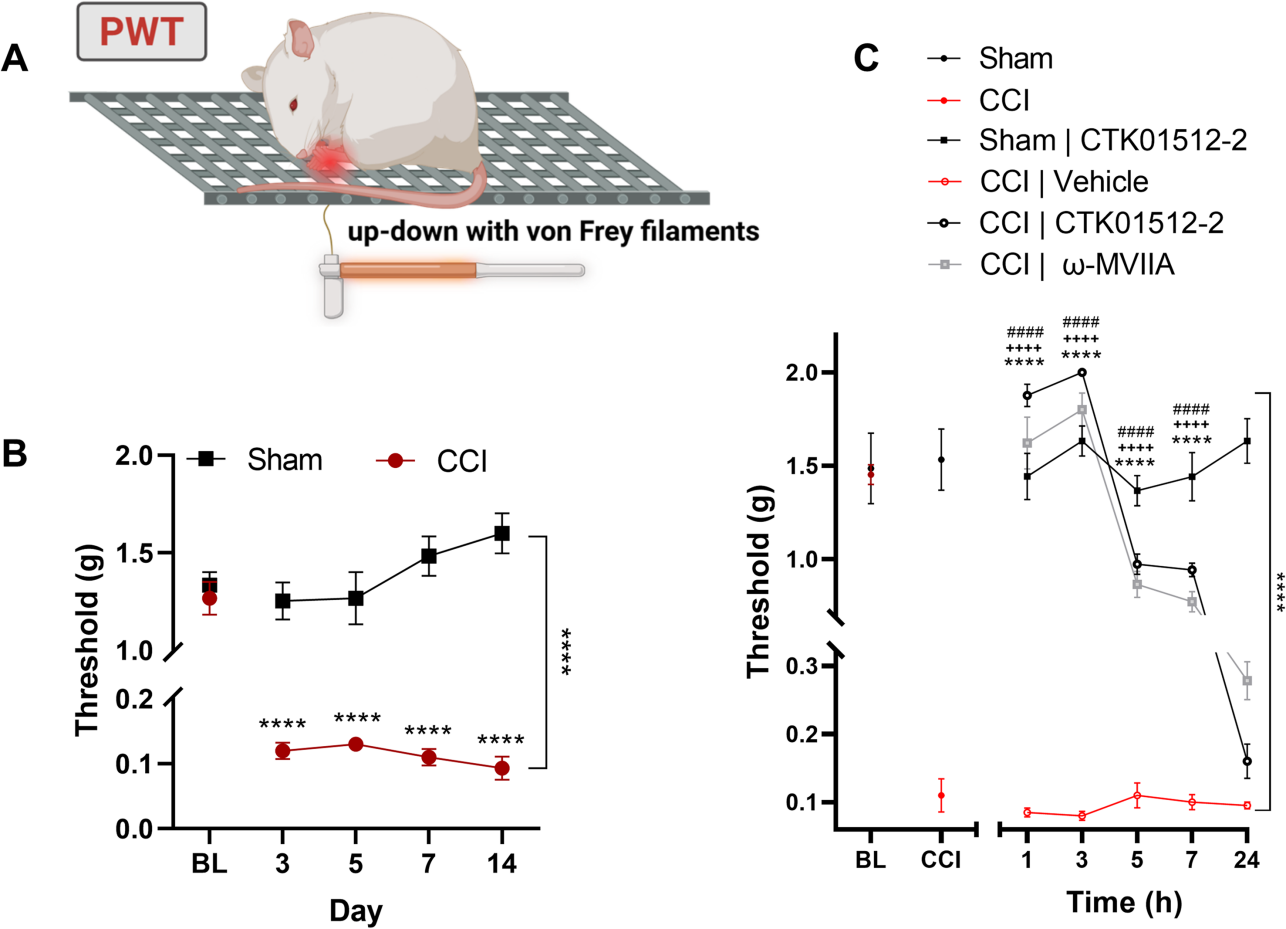
Recombinant Phα1β toxin and MVIIA reduce mechanical allodynia in the CCI model. (A) Schematic representation of the von Frey test setup. Mechanical sensitivity was assessed on the paw ipsilateral to the sciatic nerve injury (CCI side). (B) Mechanical allodynia was evaluated at baseline (BL) and on postoperative days 3, 5, 7, and 14 following CCI of the sciatic nerve. A significant reduction in mechanical withdrawal thresholds was observed at all postoperative time points compared with baseline, confirming the development of neuropathic pain (***p < 0.0001). (C) Effects of recombinant Phα1β toxin (CTK01512‐2) and MVIIA on mechanical allodynia, measured on postoperative day 14. Both treatments significantly increased paw withdrawal thresholds from the first to the seventh hour after intrathecal administration, indicating antinociceptive efficacy. Comparisons were made between CCI‐vehicle and Sham‐CTK (*), CCI‐vehicle and CCI‐CTK (+), and CCI‐vehicle and CCI‐MVIIA (#). Data are expressed as mean ± SEM (*n* = 6 per group). Due to small SEM in some conditions (e.g., CCI) *Y*‐axis is segmented and the bottom segment is scaled for optimizing visualization. The number of symbols (*) indicates the level of statistical significance: ****p < 0.0001, ***p < 0.001, **p < 0.01, *p < 0.05. Statistical analysis was performed using two‐way repeated measures ANOVA followed by Sidak's (B) and Dunnett's (C) post hoc test.

To evaluate the effects of Phα1β toxin and MVIIA on nociceptive thresholds in a neuropathic pain model, mechanical allodynia was assessed using von Frey filaments, as illustrated in the schematic (Figure 1A), at baseline and on postoperative days 3, 5, 7 and 14. As shown in Figure 1B, CCI surgery produced a robust and sustained decrease in paw withdrawal threshold compared with the sham group, confirming the successful induction of neuropathic pain. Statistical analysis revealed significant main effects of drug treatment (*F*(1, 20) = 520.20, *p* < 0.0001) and time (*F*(3, 20) = 2087, *p* < 0.0001), with no significant interaction between these factors (*F*(3, 20) = 2.665, *p* = 0.0756). From postoperative day 3 onward, groups were already significantly different, and this difference was maintained through day 14 (*p* < 0.0001 for all time points), at which point pharmacological treatments were initiated. After confirming the establishment of mechanical allodynia, the analgesic effects of CTK01512‐2 and ω‐conotoxin MVIIA were evaluated on postoperative day 14 (Figure 1C). Both treatments significantly increased the paw withdrawal threshold in CCI animals compared with vehicle‐treated controls, indicating effective reversal of mechanical allodynia. Statistical analysis revealed significant main effects of drug treatment (*F*(3, 100) = 355.9, *p* < 0.0001), time (*F*(4, 100) = 91.60, *p* < 0.0001) and a significant drug × time interaction (*F*(12, 100) = 32.78, *p* < 0.0001), indicating that antinociceptive responses varied over time. Notably, both CTK01512‐2 and MVIIA produced peak analgesic effects between 1 and 5 h after intrathecal administration, with efficacy progressively declining by 24 h.

### 3.2 Cav2.2 Calcium Currents and Their Modulation in HEK‐293 Cells

**FIGURE 2:**
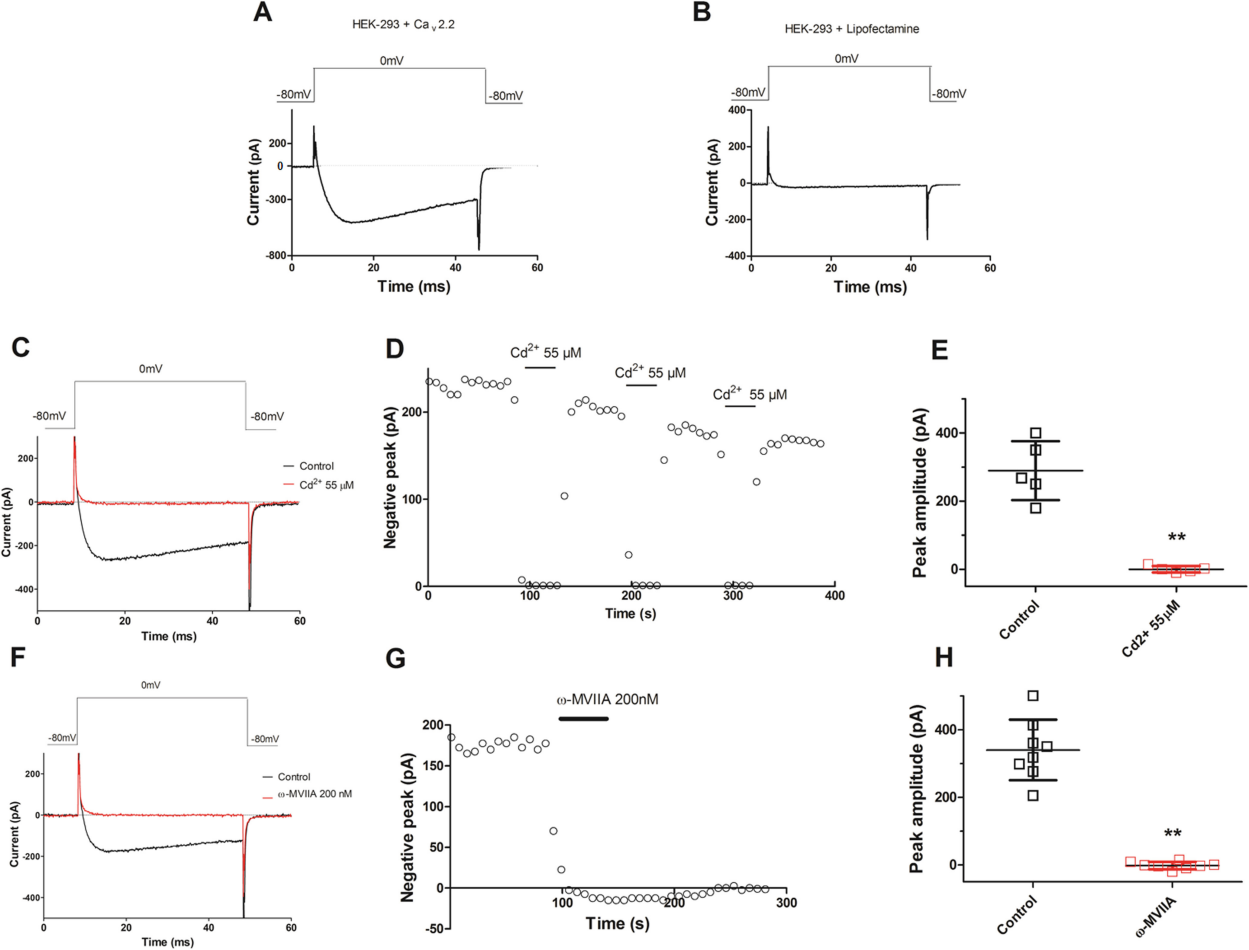
Cav2.2‐mediated currents in a transient transfection model using human embryonic kidney (HEK‐293) cells. (A) Cells were transiently transfected with plasmids containing cDNA encoding the N‐type channel (Cav2.2), composed of α1b, β3, and α2δ subunits. (B) Absence of Cav2.2‐mediated currents in cells transfected with lipofectamine alone. The recorded traces were elicited by voltage steps from a resting potential of −80 to 0 mV for 20 ms. (C,D) Cav2.2‐mediated currents in the presence of cadmium (Cd^2+^). (C) Black dashed trace: current recorded under control conditions, evoked by a voltage step from −80 to 0 mV for 20 ms. Red trace: complete inhibition of the current upon perfusion with 55‐μM Cd^2+^. (D) Plot of the negative peak amplitude of Cav2.2 currents over consecutive stimuli (7‐s interval), comparing the absence and presence of 55‐μM Cd^2+^, demonstrating total inhibition followed by current recovery after Cd^2+^ washout. (E) Quantitative plot (with mean and SEM) of the peak amplitude of currents in the absence (Control) and during Cd^2+^ exposition (*N* = 5). (F,G) Cav2.2‐mediated currents in the presence of the 200‐nM ω‐conotoxin MVIIA (ω‐MVIIA). (F) Black trace: current recorded under control conditions, elicited by a voltage step from −80 to 0 mV for 20 ms. Red trace: complete inhibition of the current upon perfusion with 200‐nM ω‐MVIIA. (G) Plot of the negative peak amplitude of Cav2.2 currents in the presence of 200‐nM ω‐MVIIA, showing total inhibition which is not retrieved after washout. (H) Quantitative plot of the peak amplitude of currents in the absence (Control) and during ω‐MVIIA exposition (with mean and SEM, *N* = 8) **p < 0.01, paired Student *t* test.

Initially, the efficiency of transient transfection was assessed. For this purpose, HEK‐293 cells were transfected with cDNA plasmids encoding the N‐type calcium channel (Cav2.2), comprising the α1b, β3 and α2δ subunits. As a negative control, cells were transfected solely with Lipofectamine, the transfection reagent, in the absence of coding cDNA plasmids. To evoke calcium currents, cells were voltage‐clamped and depolarized from a resting potential of −80 to 0 mV for 40 ms, as illustrated in Figure 2A,B. Upon transfection and voltage activation, cells exhibited an inward calcium current (311 ± 56 pA, *n* = 6 cells), indicative of calcium influx through the transfected Cav2.2 channel (Figure 2A). In contrast, cells transfected only with Lipofectamine did not display an inward current (4 ± 3 pA, *n* = 6 cells, *p* < 0.0001 Student *t* test), confirming the absence of functional channel expression in the membrane (Figure 2B). To evaluate both the responsiveness of the N‐type channel to reversible inhibition and the efficiency of perfusion, cadmium ion (Cd^2+^), a potent reversible blocker of voltage‐sensitive calcium channels (VSCCs), was applied. Perfusion of an external solution containing 55‐μM Cd^2+^ resulted in complete blockade (100%) of the Cav2.2‐mediated calcium current (Figure 2C–E). Figure 2D demonstrates the total and reversible inhibition of calcium currents upon 5‐s exposure to Cd^2+^. Upon washout of the cadmium‐containing solution, calcium currents recovered to near‐initial values (Figure 2D). These results confirm that Cd^2+^ induces a total and reversible blockade of Cav2.2‐mediated calcium currents in the transient transfection model using HEK‐293 cells. Unlike cadmium, which non‐specifically inhibits calcium currents, ω‐conotoxin MVIIA is a highly selective and potent blocker of N‐type calcium channels (Cav2.2). Therefore, ω‐conotoxin MVIIA was employed as a positive control to further assess channel function. As shown in Figure 2E,F,H, application of ω‐conotoxin MVIIA at 200 nM resulted in complete (~100%) inhibition of Cav2.2‐mediated calcium currents. The blockade induced by ω‐conotoxin MVIIA was irreversible within the experimental timeframe; even after 250 s of washout with toxin‐free external solution, no recovery of the calcium current was observed (Figure 2G). Thus, ω‐conotoxin MVIIA effectively inhibited voltage‐gated N‐type calcium currents in the transient transfection model, demonstrating its specificity and potency. These findings validate the transient transfection approach for studying Cav2.2 channel activity and pharmacological modulation.

### 3.3 Partial and Reversible Inhibition of Cav2.2 Calcium Currents by the Recombinant Phα1β Toxin (CTK01512‐2) and Its Dose–Response Relationship

**FIGURE 3:**
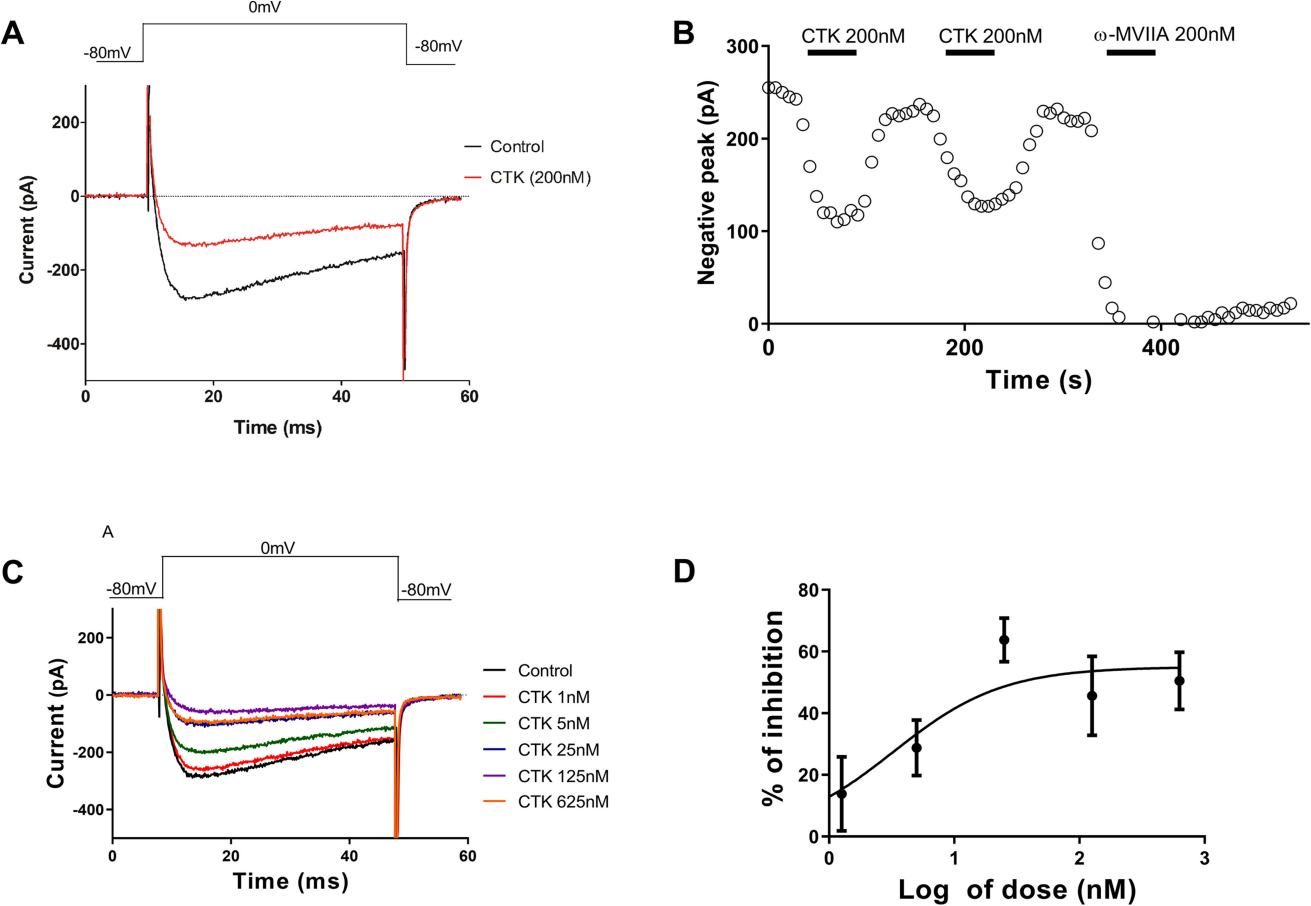
Partial reversible and dose dependent inhibition of Cav2.2‐mediated currents by CTK01512‐2. (A,B) Cav2.2‐mediated currents in the presence of the recombinant toxin Phα1β (CTK01512‐2). (A) Black trace: current recorded under control conditions, elicited by a voltage step from −80 to 0 mV for 40 ms. Red trace: partial inhibition of the same current upon perfusion with 200 nM Phα1β recombinant toxin (CTK01512‐2). (B) Plot of the negative peak amplitude of Cav2.2‐mediated currents in the presence of CTK01512‐2, showing reversible and partial inhibition. Remaining current were totally inhibited by subsequent perfusion with ω‐MVIIA. (C,D) Dose‐effect relationship of the recombinant toxin CTK01512‐2 on Cav2.2‐mediated currents. Currents were evoked by depolarization from a resting potential of −80 to 0 mV for 20 ms. (C) Black trace: control condition. Red trace: current recorded in the presence of 1 nM CTK01512‐2 Green trace: current recorded in the presence of 5 nM CTK01512‐2. Blue trace: current recorded in the presence of 25 nM CTK01512‐2. Purple trace: current recorded in the presence of 125 nM CTK01512‐2. Orange trace: current recorded in the presence of 625 nM CTK01512‐2. (E) Dose–response curve showing the inhibitory effect of CTK01512‐2 (5–625 nM) on Cav2.2 currents. IC_50_ = 3.94 nM (95% confidence interval: 0.67–23.13 nM).

The recombinant Phα1β toxin (CTK01512‐2), at a concentration of 200 nM, induced a partial blockade of the Cav2.2‐mediated calcium current (Figure 3A). This blockade was both partial and reversible (Figure 3B). Perfusion of the cell with CTK01512‐2 resulted in a reduction in the amplitude of the voltage‐activated calcium current. Figure 3A illustrates the calcium current amplitudes recorded from a representative cell before and during toxin application, 495 and 165 pA, respectively, revealing, therefore, a 65% inhibitory effect. Upon washout of CTK01512‐2 and subsequent perfusion with toxin‐free external solution, the calcium current recovered to near‐initial values (Figure 3B). Following a second washout period, incubation of the cells with 200‐nM ω‐MVIIA toxin led to a total inhibition of calcium currents, providing thus a positive control on the same cell that was previously incubated with CTK01512‐2 (Figure 3B). The dose‐dependency of the inhibitory effect of CTK01512‐2 on Cav2.2 currents was also evaluated. Depolarizing currents (−80 to 0 mV; 40‐ms duration, one step every 7 s) were elicited to cells while increasing concentrations of CTK01512‐2 were applied (Figure 3C,D). To construct the dose–response curve, concentrations of 1, 5, 25, 125 and 625 nM of CTK01512‐2 were tested. The half‐maximal inhibitory concentration (IC50), calculated from a sample of six cells, was determined to be 3.9 nM (0.7 to 23.1; 95% confidence interval) (Figure 3D).

**FIGURE 4:**
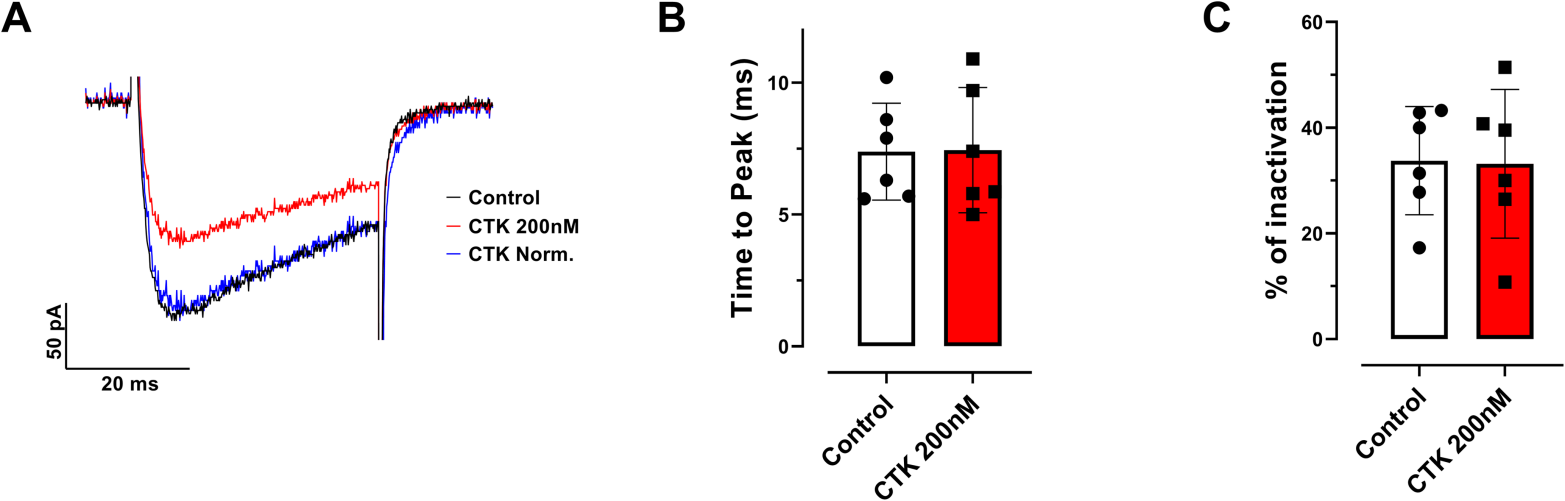
Effect of CTK01512‐2 on the kinetics and voltage‐dependence of Cav2.2 channels. (A) Representative current trace during a voltage step from −80 to 0 mV obtained under control conditions (black trace) and in the presence of CTK01512‐2 (red trace). To compare the kinetics of activation and inactivation, the trace obtained with CTK01512‐2 was scaled to match the peak amplitude of control current (blue trace). (B) Latency to peak negative current measured from the onset of the voltage step. (C) Fraction of inactivation measured at the end of the voltage step (bars represent mean ± SEM, *n* = 6, *p* > 0.05, Student paired *t* test).

To determine if CTK01512‐2 affected channel gating kinetics, we examined the time course of current activation and inactivation (Figure 4). In response to a step depolarization from −80 to 0 mV, Ca^2+^ currents activated rapidly and then slowly inactivated (Figure 4A). A similar time course was observed after treatment with CTK01512‐2, which reduced the current but did not produce a noticeable change in the kinetics of activation or inactivation. Indeed, after rescaling the current recorded in the presence of toxin to restore its peak amplitude, the control current and the scaled treated current were virtually indistinguishable (Figure 4A, blue line). To quantify these observations, we measured the latency to peak inward current after the onset of the voltage step and the degree of inactivation at the end of the 40 ms depolarization. Neither parameter was significantly affected by the toxin (Figure 4B,C).

**FIGURE 5:**
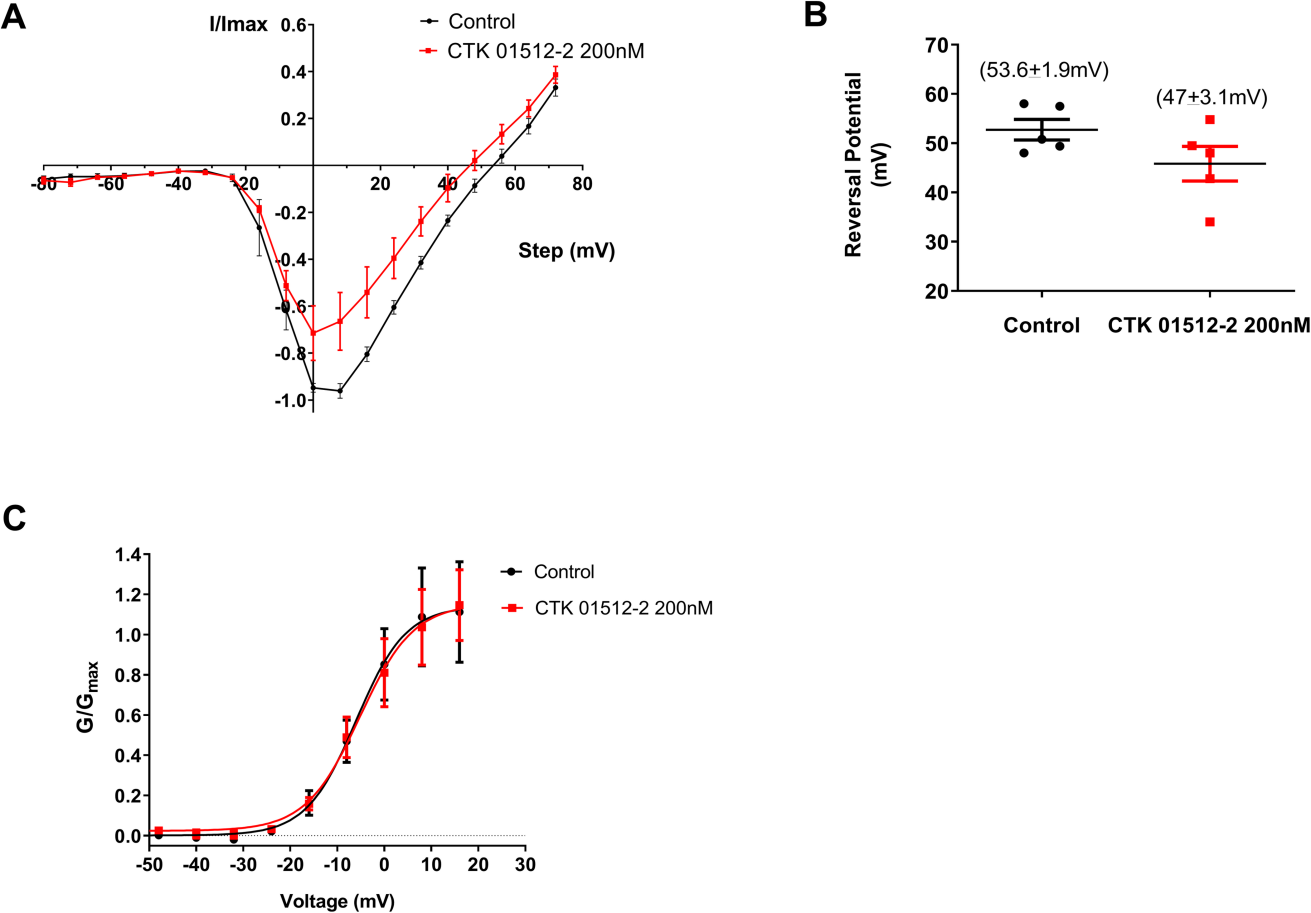
Current–voltage relationship and analysis of the activation status of the Cav2.2 calcium channel. (A) Current–voltage relationship recorded in the absence (black trace) and after incubation with 200‐nM CTK01512‐2 (red trace). (B) Reversal potential of control (black) currents and after treatment with CTK01512‐2 (Red, *p* = 0.03, Student paired *t* test). (C) Voltage‐dependent activation of Cav2.2 is not affected by 200‐nM CTK01512‐2. Currents were evoked by depolarizing voltage steps from −80 to +72 mV. The data were fitted using the Boltzmann equation: *G*/*G*_max_ = [1 = exp(*V* − *V*_h_)/*k*] − 1 where *G*/*G*_max_ represents the normalized peak conductance during depolarization, *V* is the applied voltage, *V*_1/2_ is the half‐activation voltage, and *k* is the slope factor indicating the steepness of the activation curve.

To study the voltage‐dependence of channel gating, a depolarization protocol was used starting from −80 (resting potential) to +72 mV, with increments of +8 mV at each depolarization step, as shown in Figure 5A. Calcium inward and outward currents are shown for each depolarization. For the calculation of the percentage of inhibition, we used as a reference the mean of the peak current amplitude under both conditions, recorded at 8 mV. The mean current level measured during a step to +8 mV was −414 ± 205 and −251 ± 176 pA for the control and CTK01512‐2 groups, respectively. The toxin CTK01512‐2 (200 nM) thus significantly reduced the amplitude of the calcium current by 39.4% compared with the control group mean (*p* = 0.0083).

From the *I*–*V* data, the reversal potential was determined for each cell and under each experimental condition (control and CTK01512‐2). We detected a small but significant depolarizing shift in reversal potential for currents recorded in the presence of CTK01512‐2 compared with control. This shift averaged −6.9 ± 2.2 mV (*p* = 0.03, *N* = 5).

### 3.4 Effect of CTK01512‐2 on the Voltage‐Dependence of Activation of Cav2.2 Currents

Cav2.2 calcium channels, like other voltage‐sensitive channels, exhibit voltage‐dependent activation. To investigate whether CTK01512‐2 affects this property, we analysed the voltage‐dependency of activation of transiently transfected Cav2.2 channels (Figure 5C). Normalized Calcium currents (I/*I*_max_) were converted to conductance by dividing by the driving force at each potential. The results indicated that the half‐maximal activation voltage (*V*_h_) in control cells was −6.0 ± 1.9 mV, while in the presence of CTK01512‐2, it was −5.2 ± 1.8 mV (*p* > 0.05, *t* test). The slope factor (*k*) was 5.3 ± 1.6 mV in control cells and 5.7 ± 1.4 mV in the presence of the toxin (*p* > 0.05, *t* test). These findings indicate that CTK01512‐2 did not significantly alter the voltage‐dependent activation of Cav2.2 currents in HEK‐293 cells, as no statistically significant differences were observed in *V*_h_ and *k* values between conditions (Figure 5C).

## 4 Discussion

A wide range of peptide toxins isolated from the venoms of predatory animals are known to interfere with cellular signalling and electrical excitability, with ion channels among their primary molecular targets [22, 23]. Among these, ω‐conotoxins—a family of peptides derived from marine molluscs—have become indispensable tools for identifying and characterizing calcium channel subtypes [24]. The discovery of ω‐conotoxin GVIA (from *Conus geographus*) and ω‐agatoxin IVA (from *Agelenopsis aperta* ) was pivotal for the identification of the Cav2.2 and Cav2.1 voltage‐gated calcium channels, respectively [25, 26]. Other examples include toxins from the venom of the spider *Phoneutria nigriventer* —such as Tx3–3, Tx3–4 and Tx3–6—which also inhibit voltage‐gated calcium channels [11, 27, 28].

However, the difficulty in purifying sufficient quantities of these toxins from venom has motivated efforts to produce them synthetically. Although this has been successful for some smaller peptides [29], chemical synthesis of longer peptides containing multiple disulfide bridges remains technically challenging. An alternative approach is the heterologous expression of recombinant peptides in bacteria [30]. In the present study, we characterized the recombinant Phα1β toxin (CTK01512‐2) for its antinociceptive effects in vivo and for its ability to antagonize N‐type calcium channels (Cav2.2) expressed in HEK293 cells. Our results demonstrate that CTK01512‐2 inhibits Cav2.2‐mediated currents in a partial and reversible manner. Upon washout, the currents returned close to baseline levels, indicating transient binding. The reversible inhibition observed here shares some pharmacological features with previous reports of the native Phα1β toxin, which produced reversible, although almost complete inhibition of multiple high‐voltage–activated calcium channel subtypes, including Cav1.2, Cav2.1, Cav2.2 and Cav2.3 [11]. Small differences in potency and maximal inhibition between recombinant CTK01512‐2 and native Phα1β may reflect subtle structural variations associated with distinct disulfide bond arrangements. In this regard, Wormwood et al. [31] demonstrated differences in disulfide connectivity patterns between recombinant and native Phα1β while preserving overall biological activity, suggesting that multiple conformational states may coexist without complete loss of function. Despite the partial inhibition of Cav2.2 channels by CTK01512‐2 seen in our study, the recombinant toxin still retains its pharmacological activity as an antinociceptive agent indicating that even a partial blockage of Cav2.2 can be associated to the in vivo analgesic effect of the molecule. It cannot be ruled out, however, the possibility of interaction of CTK01512‐2 with other targets that could contribute to its analgesic actions in vivo. As a positive control, ω‐conotoxin MVIIA effectively and irreversibly inhibited Cav2.2‐mediated currents within the 250‐s recording window, confirming the identity of the expressed channels. Although previous studies have reported reversible inhibition by MVIIA [32], this discrepancy is likely due to differences in recording duration. Reversibility was described only in longer protocols (up to 1200 s).

To further investigate the mechanism of action of CTK01512‐2, we examined whether it altered the voltage‐dependence of Cav2.2 activation. As with the native toxin [11] the recombinant version did not shift the voltage dependence or modify the activation kinetics. This supports the interpretation that CTK01512‐2 does not function as a gating modifier. Peptide toxins generally exert their effects on ion channels through one of two mechanisms: modulation of channel gating or physical blockade of the pore [33]. Gating modifiers, such as ω‐grammotoxin SIA and ω‐agatoxin IVA, shift the voltage‐dependence of activation, preventing channel opening under physiological conditions [33]. In contrast, pore‐blocking toxins—such as ω‐conotoxin GVIA and ω‐conotoxin MVIIC—bind to the extracellular vestibule of the channel and physically occlude ion flow [34, 35]. These interactions are typically voltage‐independent. Given that CTK01512‐2 did not affect the biophysical properties of channel activation and was rapidly reversible, our findings might support a pore‐blocking mechanism of action. Alternatively, CTK01512‐2 may interact with the gating mechanism to stabilize the closed state, as has been shown for the spider toxin huwentoxin‐IV [36]. The partial and reversible nature of the inhibition suggests that the toxin either binds with moderate affinity or is structurally unable to fully occlude the channel pore, resulting in reduced conductance rather than complete current suppression.

Calcium currents recorded in the presence of CTK01512‐2 exhibited a depolarizing shift in reversal potential (Figure 5A). The outward currents that generate the reversal potential are most likely not carried by Ca^2+^ and rather by outward movement of monovalent ions such as Cs^+^, which are present in our internal solution (reviewed by Hille [37]). Although we do not know the cause of the change in reversal potential, it could reflect changes to the ionic makeup of the outward monovalent ions. Regardless of the cause, the change in reversal potential does not affect the analysis of voltage dependence of activation because each current was divided by its own reversal potential measured for each cell and under each experimental condition (control or CTK01512‐2).

Pharmacological inhibition of Cav2.2 has been well established as an effective strategy for managing chronic CTK01512‐2pain [38, 39, 40]. In our behavioural experiments, both and ω‐conotoxin MVIIA significantly reversed the mechanical hypersensitivity induced by CCI. These results are consistent with prior studies showing that intrathecal administration of ω‐MVIIA can effectively suppress nociceptive transmission [39]. Similarly, CTK01512‐2 produced a time‐dependent antinociceptive effect lasting up to 7 h, in agreement with the observations reported by Rigo and colleagues [5].

Although the present study primarily focused on the biophysical and molecular characterization of Cav2.2 channel inhibition by recombinant Phα1β toxin (CTK01512‐2), safety and translational aspects have been previously investigated. Preclinical studies have demonstrated that intrathecal administration of Phα1β produces robust antinociception without significant motor impairment in rotarod or open‐field paradigms, supporting a favourable therapeutic window [3, 4]. Moreover, systemic toxicity evaluations and cardiovascular assessments reported minimal hemodynamic alterations at therapeutically relevant doses, with no evidence of major CNS depressant effects [10, 41]. Pharmacokinetic investigations indicate that, consistent with peptide‐based compounds, the toxin presents limited systemic bioavailability, which has supported the intrathecal route as the most effective delivery strategy thus far. Nevertheless, emerging formulation approaches, including nanoparticle‐based delivery systems and sustained‐release platforms, may expand the feasibility of systemic administration and improve half‐life and tissue distribution profiles. While detailed pharmacokinetic optimization falls beyond the scope of the present mechanistic study, the accumulated preclinical safety and translational data reinforce the therapeutic potential of Cav2.2 inhibition by CTK01512‐2. Although broader selectivity profiling across additional voltage‐gated ion channels would further refine the pharmacological characterization of CTK01512‐2, the present data establish its robust inhibitory action on Cav2.2 channels. Expanded ion channel panels and neuronal excitability studies represent important future directions for translational development.

Collectively, our data support the conclusion that CTK01512‐2 acts as a partial, reversible Cav2.2 channel blocker and produces analgesia in a model of neuropathic pain. The use of biologically active recombinant CTK01512‐2, alongside MVIIA as a control, reinforces the therapeutic potential of CTK01512‐2 as a novel analgesic agent.

## Funding

This work was supported by FAPEMIG (Grants: APQ‐03767‐23 and RED00097‐21 to MVG, APQ‐02045‐24 to CJCJ, APQ‐00842‐23 and APQ‐06532‐24 to CK), CAPES and CNPq (Grant: 310968/2023‐2 to CJCJ).

## Conflicts of Interest

The authors declare no conflicts of interest.

## Data Availability

The data that support the findings of this study are available from the corresponding author upon reasonable request.
